# Preimplantation genetic screening- the required RCT that has not yet been carried out

**DOI:** 10.1186/s12958-016-0171-z

**Published:** 2016-06-24

**Authors:** Raoul Orvieto

**Affiliations:** Infertility and IVF Unit, Department of Obstetrics and Gynecology, Chaim Sheba Medical Center (Tel Hashomer), Ramat Gan, Israel; Sackler Faculty of Medicine, Tel Aviv University, Tel Aviv, Israel

**Keywords:** PGS, NGS, Trophectoderm biopsy, Mosaicism, Aneuploidy

## Abstract

The utilization of trophectoderm biopsy combined with comprehensive chromosome screening (CCS) tests for embryonic aneuploidy was recently suggested to improve IVF outcome, however, not without criticisms. The ongoing discussion on the unrestricted clinical adoption of preimplantation genetic screening (PGS) has called for a proper randomized controlled trial (RCT), aiming to further evaluate the cumulative live birth rates (LBRs) following a single oocyte retrieval, utilizing all fresh and frozen embryos. Since this study seems not to appear for various reasons, we present herewith, the hypothetical required RCT based on the hitherto published literature.

After implementing data from the hitherto published literature on blastulation and aneuploidy rates, the rate of mosaicism and technical errors and implantation rates/LBRs of non-PGS day-3 and blastocyst and PGS blastocyst, we could clearly demonstrate the superiority of non-PGS embryo (day-3 and blastocyst) transfer over PGS blastocyst transfer, in terms of cumulative LBR (18.2–50 % vs 7.6–12.6 %, respectively).

We therefore believe that until the proper, non-hypothetical RCT on the efficacy of this procedure will appear, PGS should be offered only under study conditions, and with appropriate informed consents.

## Introduction

Preimplantation genetic screening (PGS) by blastomere aspiration of day 3 embryos, followed by ploidy analysis of these cells using fluorescence in situ hybridization (FISH), was clearly shown to be ineffective in improving in vitro fertilization (IVF) pregnancy rates and in reducing miscarriage rates [1–4]. Recently, the utilization of trophectoderm biopsy (day 5–6 embryos) combined with comprehensive chromosome screening (CCS) tests for embryonic aneuploidy, was suggested to improve IVF outcome [5], however, not without criticisms [6, 7].

The reintroduction of PGS, utilizing of trophectoderm biopsy combined with CCS tests for embryonic aneuploidy, was based on apparently improved ability to accurately diagnose embryonic aneuploidies without compromising its implantation potential. On the other hand, opponents have claimed that the reported improved efficacy and outcome are related to various factors [6, 7], including the favorably selected patients, whose embryos have reached the blastocyst stage, thus, excluding elderly and those with decrease ovarian reserve and the definition of pregnancy outcomes per embryo transfer, rather than by intention to treat.

Moreover, while all studies in favor of PGS have reported on LBR following the first embryo transfer after a fresh IVF cycle, a clinically more relevant is the cumulative LBR following a single ovarian stimulation and utilization of all fresh and frozen-thawed embryos after one oocyte retrieval. We therefore believe, that the ongoing discussion on the unrestricted clinical adoption of PGS should call for a proper randomized controlled trial (RCT), aiming to further evaluate the cumulative live birth rates (LBRs) following a single oocyte retrieval, utilizing all fresh and frozen embryos. Prompted by the aforementioned arguments, we will present the required hypothetical RCT based on the hitherto published literature.

### Current PGS clinical data

Several retrospective and prospective trials have reported improved clinical outcomes following PGS, utilizing of trophectoderm biopsy combined with CCS tests for embryonic aneuploidy. These RCTs and observational studies have been recently evaluated by Dahdouh et al. [8] in their meta-analysis, aiming to study whether PGS-CCS improves clinical implantation rates (IR) and sustained IR (beyond 20 weeks) compared with routine care for embryo selection in IVF cycles. Of the 29 eligible articles, only three RCTs and eight observational studies met full inclusion criteria, revealing significantly higher clinical and sustained IRs with the use of PGS-CCS in patients with normal ovarian reserve.

On the contrary, a recent analysis of national U.S. PGS data for 2011–2012 have yielded different results [9]. While more PGS than non-PGS cycles reached ET (64.2 % vs. 62.3 %), suggesting favorable patient selection bias for patients using PGS, LBRs per cycle start (25.2 % vs. 28.8 %) and per ET (39.3 % vs. 46.2 %) were significantly better in non-PGS cycles, whereas miscarriage rates were similar (13.7 % vs. 13.9 %).

### The hypothetical RCT (Fig. 1)

**Fig. 1:**
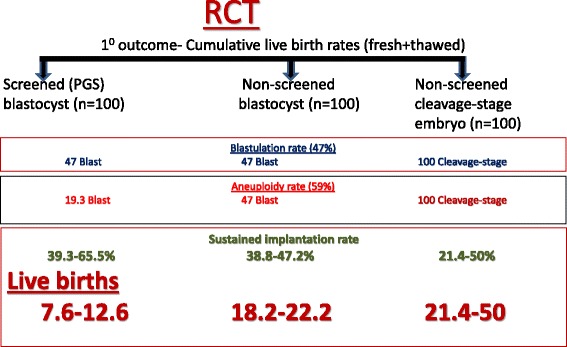
The required hypothetical RCT

The required hypothetical RCT should include 3 groups of patients with comparable clinical characteristics: those undergoing unscreened (non-PGS) day-3 transfer; those undergoing non-PGS blastocyst transfer; and those undergoing screened (PGS) blastocyst transfer.

For the propose of the analysis, we will assume that each group yielded 100 day-3 embryos and that all embryos underwent vitrification with optimal results (100 % survival post thawing).

Moreover, in order to procceed with the analysis, we need precise estimation of the following data from the hitherto published literature: blastulation rate, aneuploidy rate, the rate of mosaicism and technical errors and IRs/LBRs of non PGS day-3 and blastocyst and PGS blastocyst.

#### Blastulation rate

According to a comprehensive cochrane review that analyzed cleavage stage versus blastocyst stage embryo transfer in assisted reproductive technology [10], the range of blastocyst formation rates across studies varied between 28 and 97 %. With a mean blastulation rate of *47 %.*

#### Aneuploidy rate

Franasiak et al. [11] have presented their clinical experience while reviewing 15,169 consecutive trophectoderm biopsies evaluated with CCS. As expected, the prevalence of aneuploidy rose steadily with age. The prevalence of aneuploidy was 20 to 27 % in women 26 to 30 years of age, rose steadily from age 31 through age 43, and then plateaued at approximately 85 %. Among the biopsies with aneuploidy, 64 % involved a single chromosome, 20 % two chromosomes, and 16 % three chromosomes, with the proportion of more complex aneuploidy increasing with age. The calculated overall aneuploidy risk across all screened blastocysts was *59 %.*

#### Mosaicism and technical errors

In a recent study [12] evaluating the accuracy of trophectoderm multiple biopsies using next-generation sequencing (NGS), 5 of the 24 (20.8 %) trophectoderm biopsies revealed inconclusive results, while 4 (16.6 %) demonstrated embryonic mosaicism. When considering only NGS results without a background noise, discordant results (mosaicism) were observed in 3 out of the 8 embryos. Moreover, 3 of the 18 (16.6 %) trophectoderm biopsies were inconclusive. Overall, 8 (36.3 %) of the 22 biopsies without a background noise revealed mosaicism or inconclusive results.

In agreement with the aforementioned observation, Greco et al. [13] have recently showed that mosaic embryos can develop into healthy euploid newborns. Of 18 patients undergoing a transfer of a mosaic embryo, 6 conceived and delivered a normal euploid infant at term.

We therefore should not ignore the possibility of misdiagnosis, which may lead to false positive and false negative results. While a false positive result, such as those describe by Greco et al. [13], may reduce IVF outcome because a healthy embryo is not transferred, a false negative result, where an abnormal embryo is transferred, will lead to the delivery of an “abnormal/aneuploid” child. As a consequence of these observations, whatever the outcome following PGS blastocyst transfer would be, the LBR would be even lower due to the mosaicism and technical errors.

#### Implantation and live birth rates

A recently published CDC report [14] has presented the data regarding the percentages of day 3 and day 5 embryo transfers resulting in live births, by age group. Live birth rates following day 3 embryo transfer ranges between 23.1 and 38.5 % (crude mean 31.2 %) in patients age 38–40 vs <35 years, respectively. The corresponding figures following non-PGS blastocyst transfer were 35.5 and 52.5 % (crude mean 38.8 %) respectively.

While reviewing the effect of elective single embryo transfer compared with double embryo transfer following IVF, Min et al. revealed ongoing pregnancy/Live birth rates per cleaved embryo transfer, ranging between 21.4 and 38.5 % [15].

Moreover, the sustained implantation rate of day-3 embryo reached 50 % in Scott et al. report, while evaluating the effect of embryo biopsy on reproductive competence [16].

A close look at Dahdouh et al. [8] meta-analysis revealed a sustained IR (beyond 20 weeks) of 65.5 % for PGS-blastocyst, as compared to 47.2 % for non-PGS blastocyst, figures that are in accordance with Scott et al. [17]. The corresponding figures, as reported by the analysis of national U.S. PGS data for 2011–2012, are 39.3 % vs. 46.2 %, respectively [9].

To summarize, the reported/expected sustained IRs per day-3 non-PGS embryos, PGS and non-PGS blastocysts range between *21.4 and 50* %, *44* and *47.2 % and 39.3 and 65.5* %, respectively.

### Data analysis (Fig. 1)

Implementation of the aforementioned figures to the hypothetical RCT analysis reveals the highest LBR in patients undergoing non-PGS day 3 embryo transfer (21.4–50 %), followed by Non-PGS blastocyst (18.2–22.2 %). Patients undergoing PGS blastocysts transfer achieved the lowest LBR (7.6–12.6 %).

## Conclusions

In the present hypothetical RCT, we clearly demonstrated the superiority of non-PGS embryo transfer over PGS blastocyst transfer, in terms of cumulative LBR (18.2–50 % vs 7.6–12.6 %, respectively). These figures are in accordance with the recently raised debate [17] regarding the risks and benefits of extended embryo culture. Maheshwari et al. [18] have demonstrated that although blastocyst transfer results in higher LBRs per embryo transfer episode, it ultimately results in lower cumulative live birth rates per couple, higher risk of preterm birth, large for gestational age, monozygotic twins and congenital anomalies compared with embryo transfer at cleavage stage.

The present analysis adds further information to the hitherto published data and may contribute to the ongoing discussion on the unrestricted clinical adoption of PGS, that we believe that until the proper, real, non-hypothetical RCT on the efficacy of this procedure will appear, PGS should be offered only under study conditions, and with appropriate informed consents.

## Abbreviations

CCS, comprehensive chromosome screening; FISH, fluorescence in situ hybridization; IVF, in vitro fertilization; LBR, live birth rate; NGS, next-generation sequencing; PGS, preimplantation genetic screening; RCT, randomized controlled trial
